# Supplementary material for: X10 expansion microscopy enables 25‐nm resolution on conventional microscopes
Source: EMBO Rep. 2018 Jul 10;19(9):e45836. doi: 10.15252/embr.201845836 (PMC6123658; doi:10.15252/embr.201845836)
Supplement: Supplementary file 1 — Appendix [file EMBR-19-e45836-s001.pdf]

APPENDIX

for

X10 Expansion Microscopy Enables 25 nm Resolution on Conventional Microscopes

TABLE OF CONTENTS

APPENDIX FIGURES 2

Appendix Figure S1: At sufficient resolution, microtubules appear discontinuous with conventional antibody stainings. 2

Appendix Figure S2: Simulations of Tubulin imaging describe expansion microscopy resolution limits. 3

Appendix Figure S3: An analysis of antibody staining in X10 microscopy. 5

Appendix Figure S4: Comparison of presynaptic active zone and postsynaptic density separation. 6

Appendix Figure S5: Analysis of X10 results in rat brain slices. 9

Appendix Figure S6: Disruptions in Mechanically Tougher Tissues. 11

APPENDIX REFERENCES 12

APPENDIX FIGURES

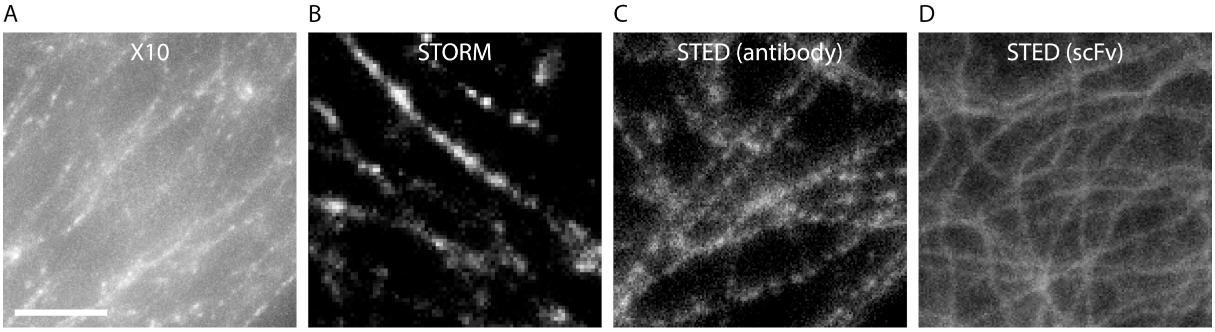

Appendix Figure S1: At sufficient resolution, microtubules appear discontinuous with conventional antibody stainings.

(A) An image of microtubules, after a staining with conventional primary and secondary antibodies, as described in the Methods, after X10 expansion. Scale bar: 1 µm; also applies to (B-D).

(B-D) Comparable images of microtubules, imaged on a STORM setup and a STED setup (with an antibody staining, and with a single chain variable fragment, scFv, staining). It becomes apparent that the microtubules are discontinuous, showing a pearls-on-a-string pattern, in all cases. The highest staining density is provided by the scFv staining, which is expected, due to the lower size of this probe [1].

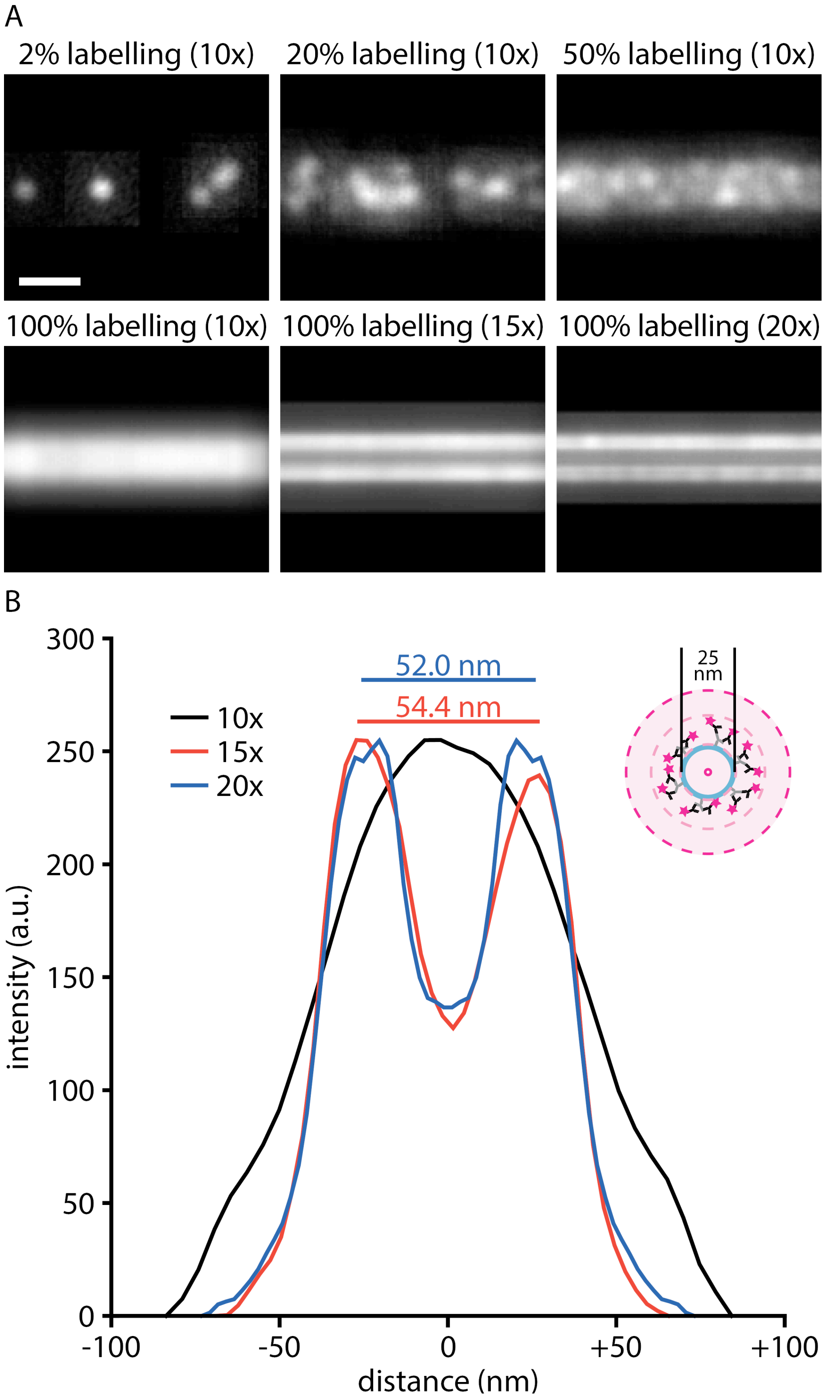

Appendix Figure S2: Simulations of Tubulin imaging describe expansion microscopy resolution limits.

(A) Simulations of a microtubule with increasing labelling densities of primary antibody (2%, 20%, 50%, 100%), and at increasing expansion factors for maximal labelling (10x, 15x, 20x). Low labeling densities result in punctate, discontinuous microtubules, as already indicated in Appendix Figure S1. This implies that optimal labelling density is critical for the interpretation of structures in expansion microscopy, as in super-resolution imaging in general. Scale bar: 100 nm.

(B) Line scans across the simulated microtubule images with maximal labelling reveal that an expansion factor of at least 15x is necessary to resolve the microtubule lumen. At these expansion factors the displacement of fluorescent probes by the primary/secondary antibody

complexes also has to be taken into account (see inset, a model of a microtubule decorated with primary/secondary antibody complexes with point-spread-functions as they would appear after 10x expansion, after the fashion of the model shown in Figure S4A). The observed size of the structures under investigation no longer fits with the actual size of the respective structures. The microtubule diameter is 25 nm, but appears to be ~55 nm in a 20x expansion image (see also [2]).

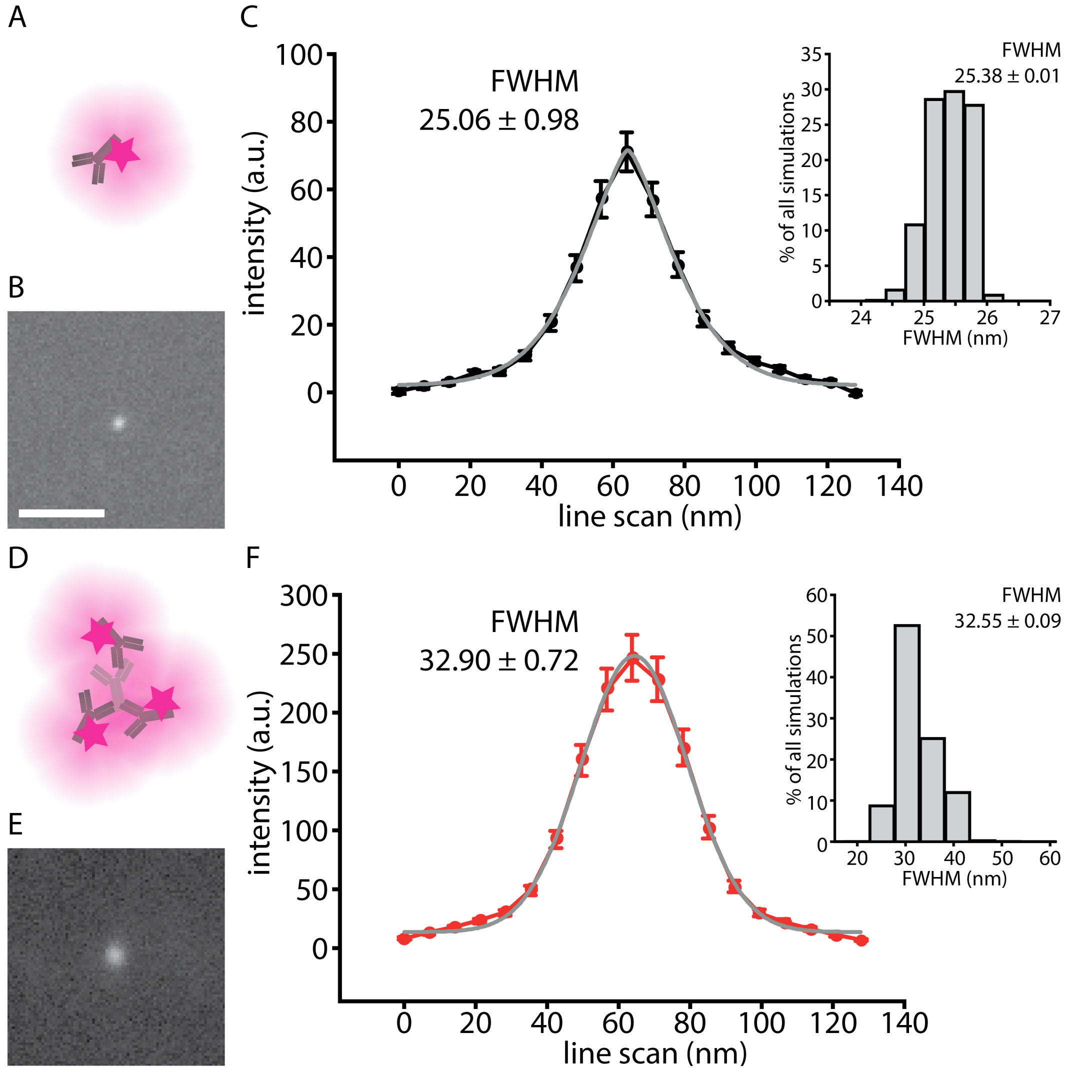

Appendix Figure S3: An analysis of antibody staining in X10 microscopy.

(A-B) Fluorescently-conjugated antibodies (anti-mouse antibodies conjugated to Alexa 488) were added onto coverslips, were fixed, and were expanded, before analysis by epifluorescence microscopy. Scale bar: 100 nm.

(C) Single antibodies were imaged, and line scans were manually drawn on individual spots, before averaging (n = 57), and fitting a Gaussian curve (gray line). The FWHM and standard error of the fit are shown on the graph. To test whether this value is realistic, we modeled this experiment as in Figure S4. The resulting values are shown in the histogram in the inset, from more than 2000 simulations.

(D-E) Same as panels A-B, but in an experiment in which unconjugated mouse antibodies (anti-Synaptotagmin 41.1 antibodies) were first applied onto coverslips, before secondary labeling with anti-mouse antibodies conjugated to Alexa 488.

(F) Same as panel C, for the secondary-labeled antibodies (n = 125). The intensity of the spots confirms that they consist, on average, of a primary antibody conjugated to 3 secondary antibodies (as the intensity is roughly 3-fold higher than that obtained in panel C). The Gaussian fit also shows a larger FWHM, which was confirmed by simulations (inset).

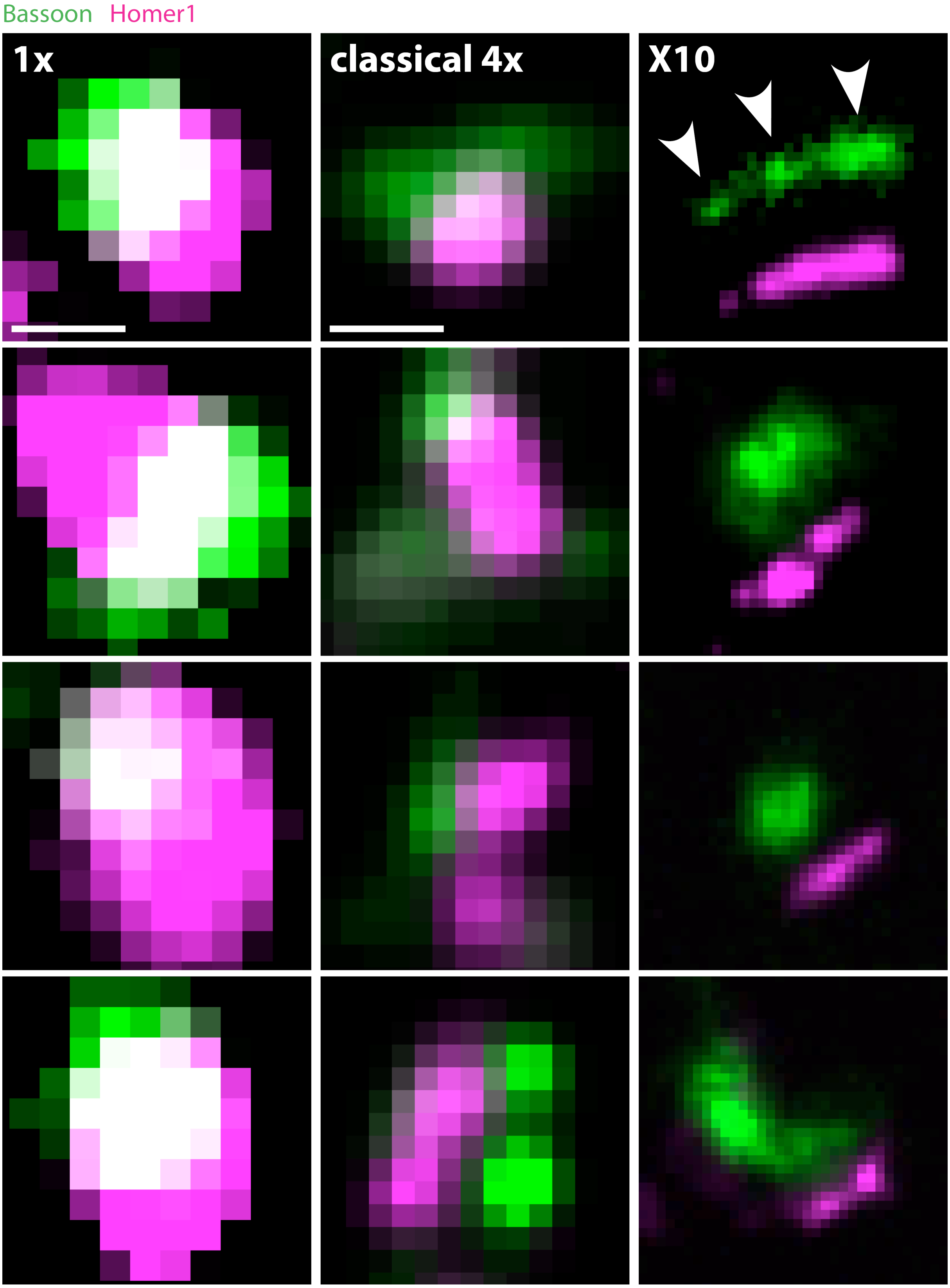

Appendix Figure S4: Comparison of presynaptic active zone and postsynaptic density separation.

Comparison of presynaptic active zone and postsynaptic density separation in conventional imaging (1x), "classical" expansion microscopy (4x), and X10 microscopy (10x). Only X10 microscopy supplies a resolution that is sufficient for a proper analysis of these sub-cellular structures (same image as shown in the example in Figure 3B,D, for comparison with the line

scans depicted there). Expansion factors: 4.0x and 11.0x. Scale bar for 1x: 400 nm. Scale bar for 4x and X10: 200 nm.

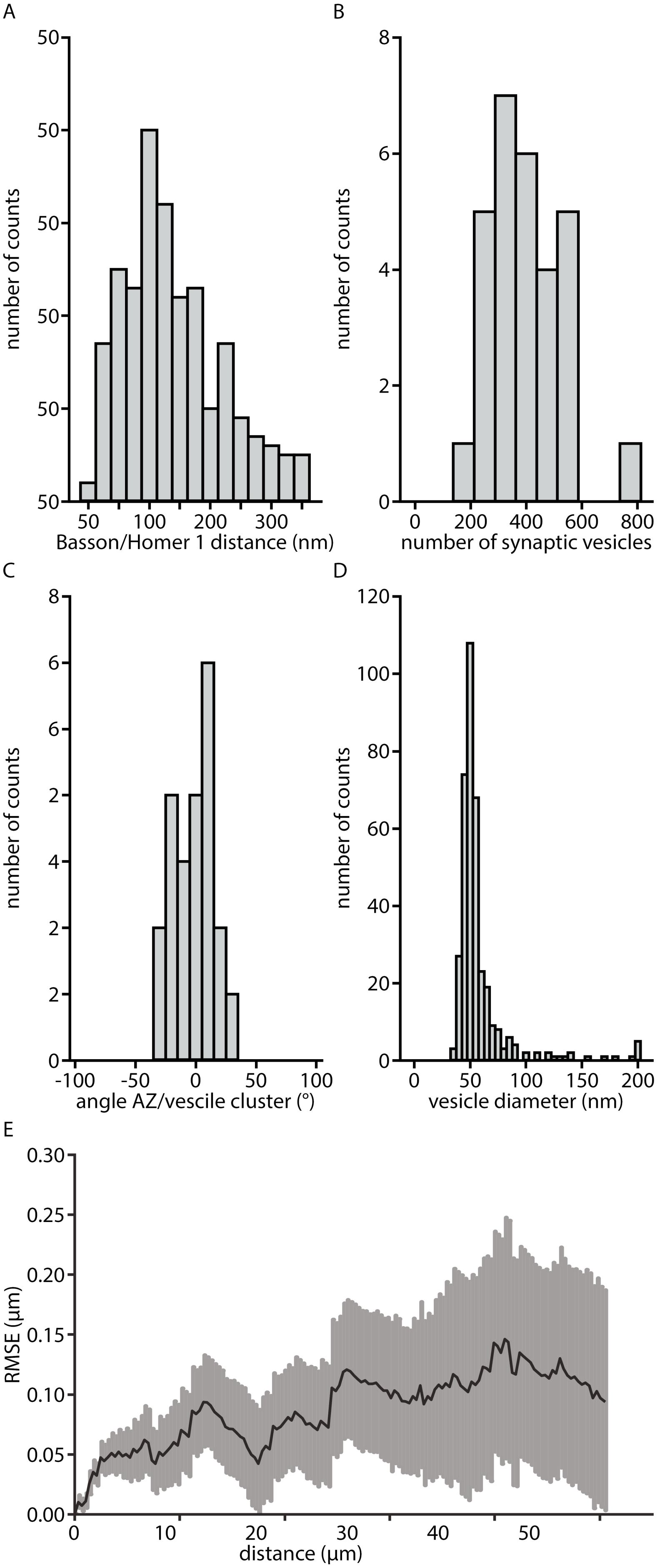
A
B
C
D
E
number of counts
Basson/Homer 1 distance (nm)
50
100
200
300
0
2
4
6
8
200
400
600
800
number of synaptic vesicles
angle AZ/vescile cluster (°)
-100
-50
0
50
100
vesicle diameter (nm)
20
40
60
80
100
120
150
RMSE (µm)
0.00
0.05
0.10
0.15
0.20
0.25
0.30
distance (µm)
10
30
40

Appendix Figure S5: Analysis of X10 results in rat brain slices.

(A) We analyzed the distance between Bassoon and Homer protein clusters, as in Fig. 4 (n = 243 distances, from 16 slice areas containing synapses). The values are similar to those obtained in cell cultures (Fig. 4).

(B) We analyzed the number of synaptic vesicles (identified by Synaptophysin) in brain slices (n = 29 different synapses that could be easily identified). The value is similar to values obtained previously by electron microscopy in the same brain region we analyzed, where an average of 480±160 vesicles had been found from 22 fully reconstructed synapses [3].

(C) We analyzed the positioning of the active zone, marked by Bassoon, in relation to the center of the synapses (marked by the center of the Synaptophysin staining), for neighboring synapses, in several Z-stacks where multiple neighboring synapses could be detected (n = 22). We calculated the angle made by the line drawn through the Bassoon spots and the synapse center for each synapse, and subtracted from it the average angle obtained from all synapses in the same image. If the synapses are randomly oriented, the resulting measurement varies randomly. In reality the values were clustered around 0°, indicating that the Bassoon spots tended to be oriented in the same fashion in relation to the synapse centers, in each of the fields analyzed.

(D) We analyzed synaptic vesicle sizes in brain slices by generating line scans over the Synaptophysin spots automatically. These were fitted with Gaussian curves, from which we determined the resulting vesicle diameters (FWHMs). These peak at 50 nm, which is in line with the vesicle sizes, taking into account that the original diameter of the vesicle (42 nm) is somewhat blurred by the antibody staining, due to the antibody size (n = 372).

(E) An analysis of the root mean square error (RMSE) of the distortions between aligned pre- and post-expansion images in brain slices (n = 19 automated measurements from 2 independent experiments).

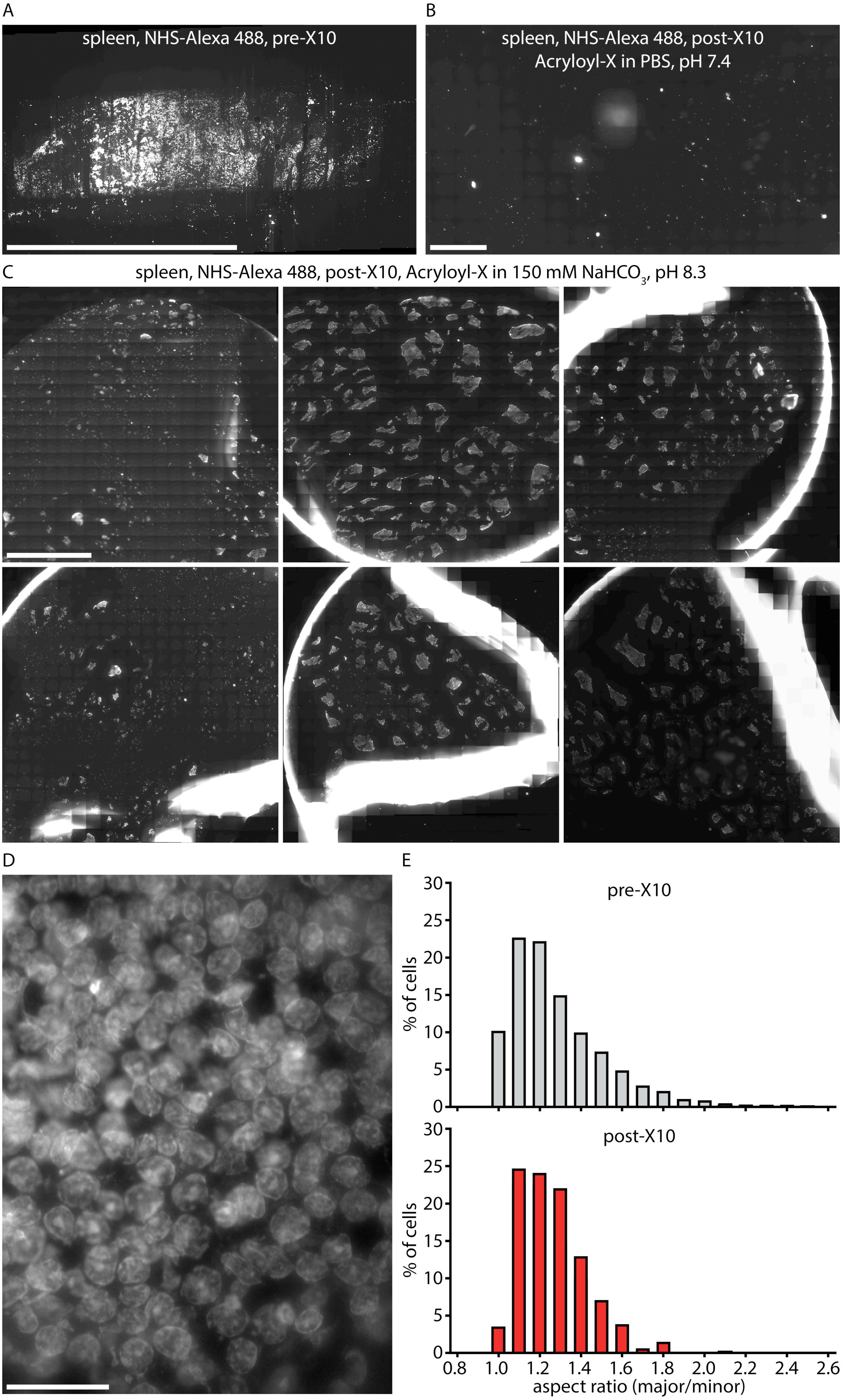

A
spleen, NHS-Alexa 488, pre-X10
B
spleen, NHS-Alexa 488, post-X10
Acryloyl-X in PBS, pH 7.4
C
spleen, NHS-Alexa 488, post-X10, Acryloyl-X in 150 mM NaHCO3, pH 8.3
D
E
pre-X10
post-X10
% of cells
0
5
10
15
20
25
30
0.8
1.0
1.2
1.4
1.6
1.8
2.0
2.2
2.4
2.6
aspect ratio (major/minor)

Appendix Figure S6: Disruptions in Mechanically Tougher Tissues.

(A) Mouse spleen tissue section, labelled with NHS-Alexa 488, before expansion. Scale bar: 1 cm.

(B) Mouse spleen tissue section, labelled with NHS-Alexa 488, after expansion with Acryloyl-X anchoring in PBS at pH 7.4, and overnight digestion with proteinase K at room temperature. The expansion factor cannot be determined, due to scale of disintegration, which removes most tissue material. Scale bar: 200 µm.

(C) Mouse spleen tissue sections, labelled with NHS-Alexa 488, after expansion with Acryloyl-X anchoring in 150 mM $NaHCO_3$ at pH 8.3 (to increase the efficiency of the anchoring reaction), and overnight digestion with proteinase K at 50°C, in buffers containing 2 mM $CaCl_2$ (to increase the enzyme stability and activity, and thus to increase the efficiency of the homogenization). Expansion factor: ~9.0x for all of the gel pieces imaged (from two different spleen sections). Scale bar: 500 µm.

(D) Post-X10 image of spleen tissue labeled with NHS-Alexa 488. Within the disintegrated gel, individual regions can still be seen to maintain their regional coherence on the level of cells. Expansion factor: ~8.8x. Scale bar: 10 µm.

(E) Histograms of cell aspect ratios before and after expansion show that within individual pieces of tissue, distortions are minimal; the cells maintained their overall size and shape.

APPENDIX REFERENCES

1. Mikhaylova M, Cloin BMC, Finan K, Van Den Berg R, Teeuw J, Kijanka MM, Sokolowski M, Katrukha EA, Maidorn M, Opazo F, et al. (2015) Resolving bundled microtubules using anti-tubulin nanobodies. *Nat Commun* **6**: 1–7.

2. Chang JB, Chen F, Yoon YG, Jung EE, Babcock H, Kang JS, Asano S, Suk HJ, Pak N, Tillberg PW, et al. (2017) Iterative expansion microscopy. *Nat Methods* **14**: 593–599.

3. Xu-Friedman MA, Harris KM, Regehr WG (2001) Three-dimensional comparison of ultrastructural characteristics at depressing and facilitating synapses onto cerebellar Purkinje cells. *J Neurosci* **21**: 6666–6672.
