# Supplementary material for: X10 expansion microscopy enables 25‐nm resolution on conventional microscopes
Source: EMBO Rep. 2018 Jul 10;19(9):e45836. doi: 10.15252/embr.201845836 (PMC6123658; doi:10.15252/embr.201845836)
Supplement: Supplementary file 2 — Expanded View Figures PDF [file EMBR-19-e45836-s002.pdf]

Expanded View Figures

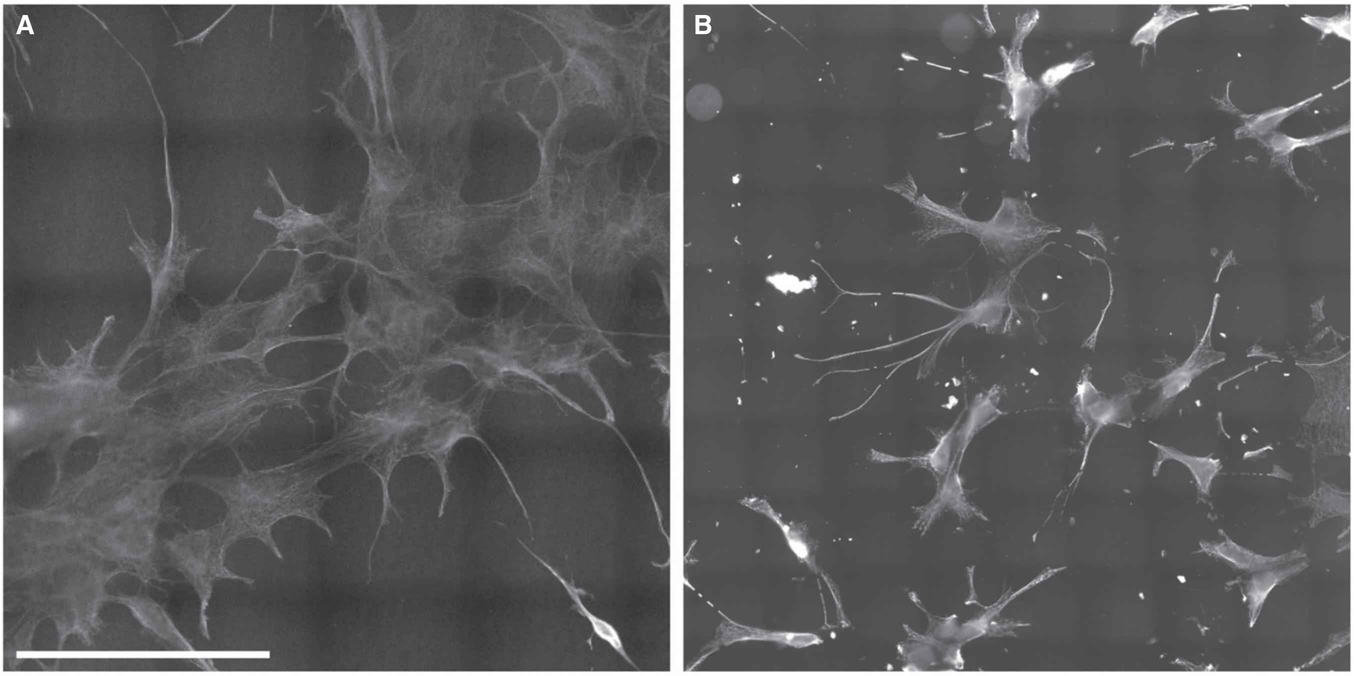

Figure EV1. Insufficient anchoring, gel polymerization, or digestion can affect the sample integrity.

A An intact sample of COS7 cell stained for tubulin, after expansion. Cell integrity is maintained, and no breaks or tears are evident. Anchoring, gel polymerization, and digestion were carried out as described in Materials and Methods. Expansion factor: 11.4×. Scale bar: 100 μm.

B A damaged sample of COS7 cells stained for tubulin, after expansion. Multiple tears and distortions are evident. This occurs when protein retention, anchoring, and gel polymerization are incomplete, i.e., because of insufficient polymerization time, or when digestion is incomplete, i.e., through digestion at room temperature instead of at 50°C. Expansion factor: 9.4×.

Data information: Note that both images are stitched together from multiple imaging frames.

© 2018 The Authors

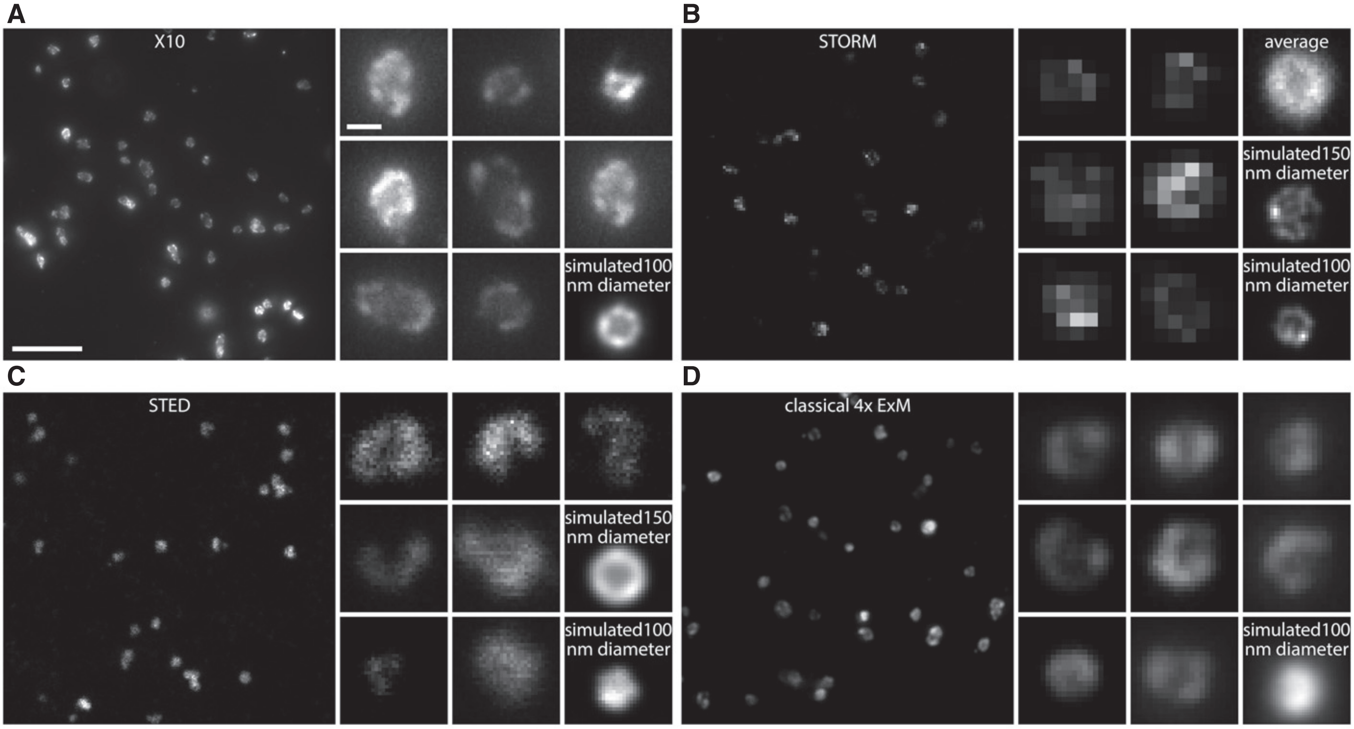

Figure EV2. X10's resolution of ~25 nm is superior to commercial state-of-the-art STED and STORM setups.

A X10 microscopy of neuronal peroxisomes stained for the peroxisome membrane marker Pmp70 (maximum intensity projections from a *z*-stack). The large panel shows an overview image, while the smaller panels are a gallery of individual peroxisomes. The left bottom panel (simulated) shows a simulated peroxisome, from a model that takes into account antibody size, as well as the random distribution and orientation of primary and secondary antibodies. Expansion factor: 9.4×. Scale bars: 1 μm (large panel), 100 nm (small panels); the scale bars also apply to (B–D). For comparison purposes, a simulated peroxisome with a diameter of 100 nm is shown.

B STORM of neuronal peroxisomes stained for the peroxisome membrane marker Pmp70. The panels are arranged as in (A), and an average peroxisome (constructed from averaging 37 peroxisomes from our experimental data) is also shown. The lower level of detail that can be reliably observed in the STORM-revealed peroxisomes is due to two effects: First, the blinking behavior of the fluorophores is not perfect, which implies that not all antibodies will be detected, resulting in a spottier pattern than in X10; and second, the entire volume of the peroxisome is within the imaging plane. For X10, the enlargement along the *z*-axis implies that only ~2/3 of the peroxisome volume is in focus, which sharpens the detection of the lateral membrane. The STORM images are readily reproduced by simulations of peroxisomes, if we take into account the factors explained above (both a 100-nm peroxisome and a 150-nm peroxisome are simulated).

C STED microscopy of neuronal peroxisomes stained for the peroxisome membrane marker Pmp70. The panels are arranged as in (A); again, two simulated images are provided, one for a peroxisome with 100 nm diameter and one for a peroxisome with 150 nm diameter. The state-of-the-art commercial STED setup that we used achieved a lateral resolution of ~38.9 nm here, which is sufficient to detect reliably the lumen of the 150-nm-diameter peroxisomes, but has more difficulties for smaller ones, such as the 100-nm-diameter peroxisome.

D Classical 4× expansion microscopy of neuronal peroxisomes stained for the peroxisome membrane marker Pmp70 (maximum intensity projections from a *z*-stack). The panels are arranged as in (A). Expansion factor: 3.8×. As for STED microscopy, the lumen of a 100-nm-diameter peroxisome is virtually impossible to discern (see simulation), but the lumen is visible in larger peroxisomes.

 © 2018 The Authors

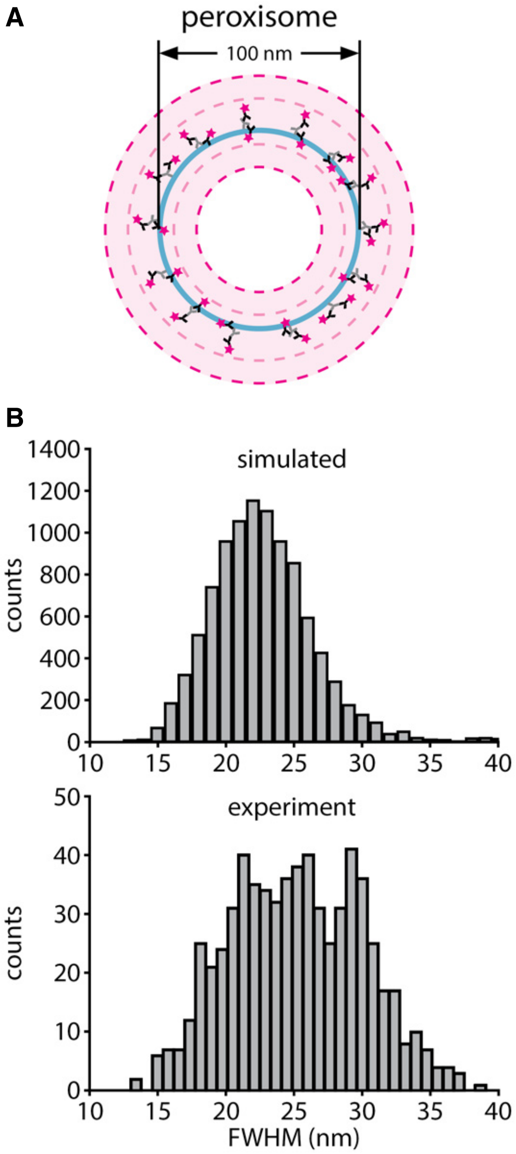

Figure EV3. A model for predicting useful resolution in expansion microscopy.

A The theory of the model. A peroxisome with a diameter of 100 nm is shown in light blue, decorated with primary antibodies (light gray) and secondary antibodies (black with magenta stars to indicate fluorophores), which are placed randomly, and with random orientations. The penetration of the secondary antibody into the peroxisome lumen is allowed, to account for the permeabilization of the peroxisome membrane during immunostaining. The dashed light magenta rings indicate the outer and inner bounds of fluorophore localizations. The dashed dark magenta rings indicate the outer and inner bounds of the diffraction-limited point spread functions resulting from the fluorophores, in conventional epifluorescence microscopy. The point spread function bounds are placed as they would appear after 10× expansion of the structure (the size bars indicated in the figure reflect the original size of the structure, not the size after expansion). It is apparent that X10 should be readily able to resolve the lumen of peroxisomes with a diameter of 100 nm.

B Histograms showing the resolution, expressed as the full width at half maximum (FWHM), measured on the membrane edges of simulated (top, 10,000 simulated line scans) and experimentally imaged (bottom, 653 line scans) peroxisomes. The model and the experimental data are in good agreement, with the model predicting an average resolution of 22.8 nm and an experimentally determined average resolution of 25.2 nm (see Fig 3).

© 2018 The Authors

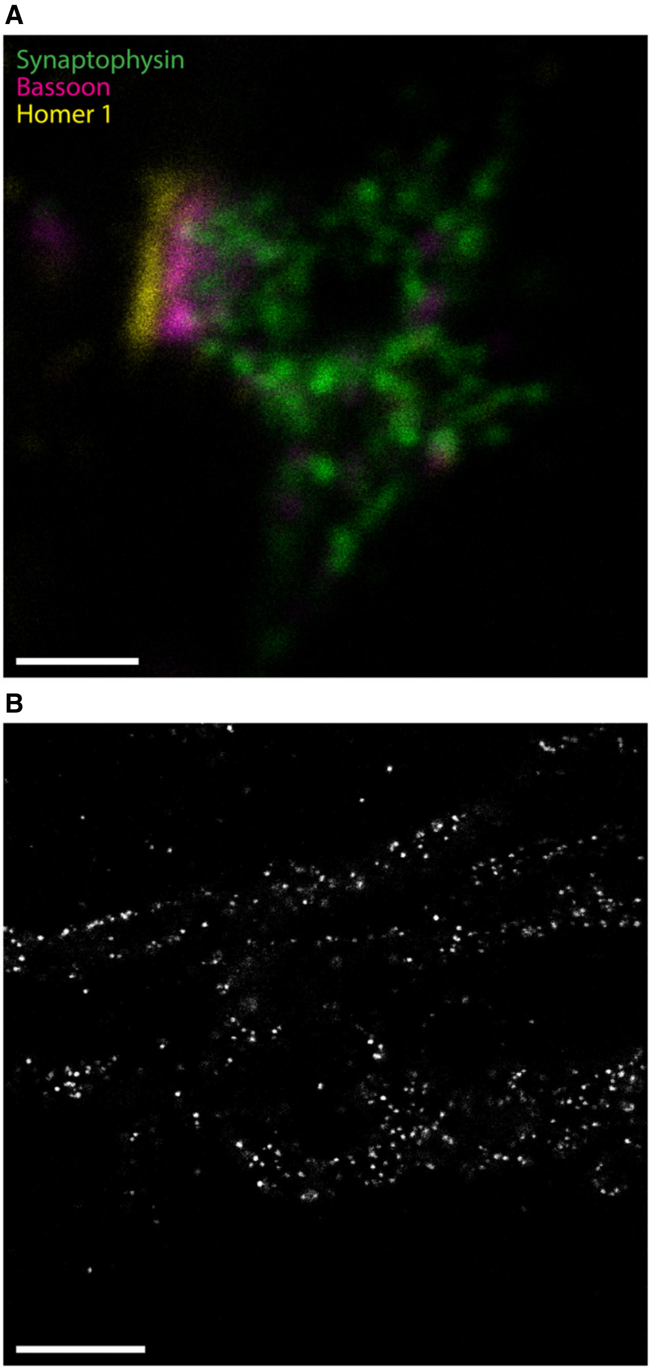

Figure EV4. Confocal imaging of synapses in X10 microscopy.

A This exemplary image shows a synapse, with staining for the same markers as in Fig 4, but imaged on a confocal microscope. The separation of pre-synaptic active zone (Bassoon, magenta) and post-synaptic density (Homer 1, yellow) is visible, and individual synaptic vesicles (Synaptophysin, green) can be distinguished. Expansion factor: 9.5×. Scale bar: 200 nm.

B This exemplary image shows mitochondria in U2-OS cells, stained for TOM20. TOM20 forms clusters in the outer membrane of mitochondria. The samples are extremely dim, since they have been expanded by ~1,000-fold in volume, which makes confocal imaging difficult, as only a few scattered “dots” are seen. Expansion factor: 10.0×. Scale bar: 1 μm.

 © 2018 The Authors

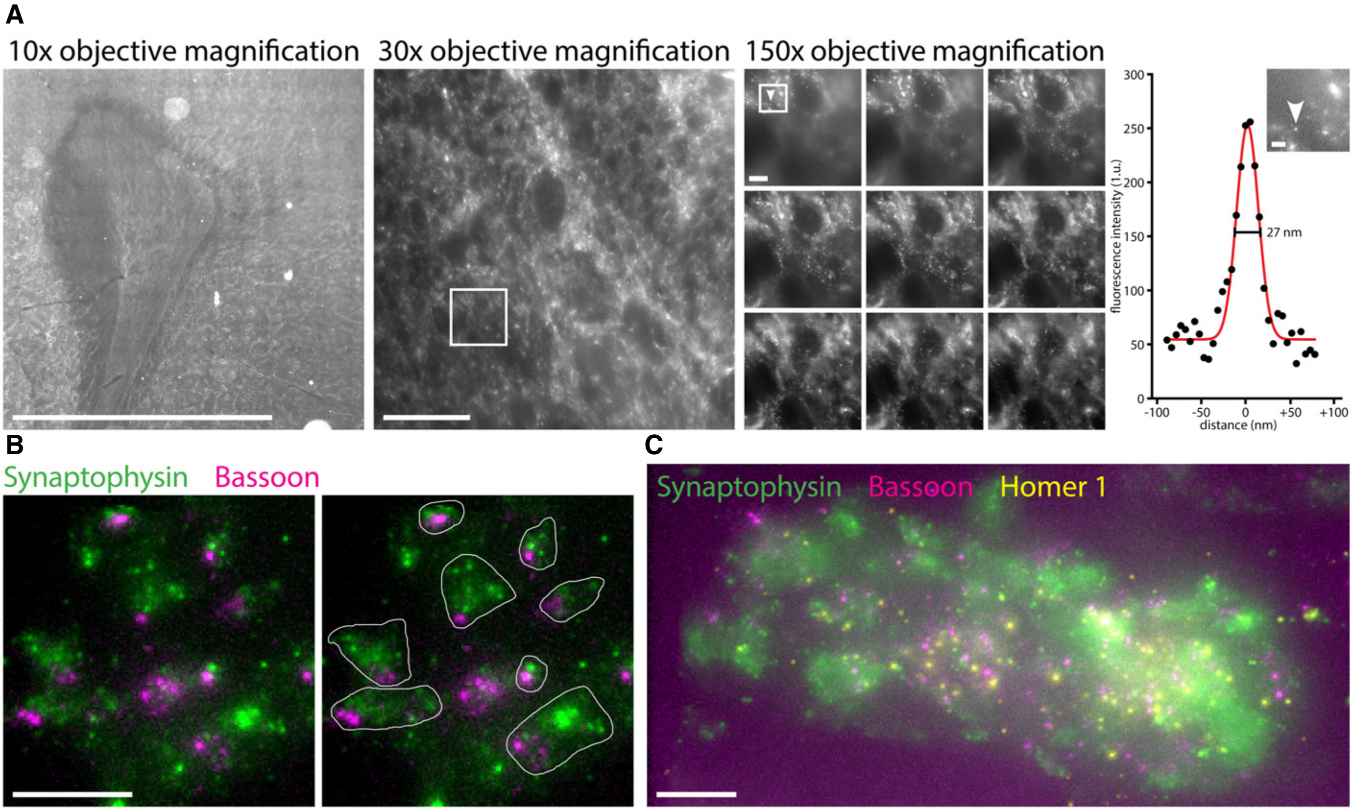

Figure EV5. Examples of X10 in overview images of rat brain slices.

A X10 microscopy in brain slices reveals the usefulness of this imaging technique across a wide range of magnifications. Macroscopic overview images can be easily collected together with super-resolution images at higher magnifications, in the same sample. Shown are a low-magnification overview image of a cortical rat brain slice stained for synapsin I/II (left, 10× microscope objective magnification, stitched together from several imaging frames), a higher magnification (middle, 30× microscope objective magnification, maximum intensity projection of a *z*-stack), and a *z*-stack of higher still magnification (right, boxed are from middle panel, 150× microscope objective magnification, *z*-stack from top left to bottom right, 9.4 μm step size). Next to the right pane is a line scan (black dots) through a small punctate structure in the brain slice, shown by the arrowhead in the inset (boxed area from the 150× magnification panel), with a best Gaussian fit (red). This reveals a FWHM resolution of 27 nm. Expansion factor: 9.4×. Scale bars: from left to right, 1 mm, 10 μm, 1 μm, 200 nm.

B Synapses in the rat cerebellum seem to be oriented in a similar direction, as seen from maximum intensity projections of *z*-stacks. In the left image, the synapses (green, Synaptophysin labeling of synaptic vesicles) tend to have their active zones (magenta, Bassoon labeling) around the bottom left area. In the right image, the synapses are marked by white outlines for easier visualization. Expansion factor: 9.6×. Scale bar: 1 μm. The position of the active zone in relation to the vesicles has been analyzed in Appendix Fig S5.

C An overview image of a larger area in a rat cerebellum brain slice in a maximum intensity projection of a *z*-stack. Multiple closely packed synapses (green, Synaptophysin labeling of synaptic vesicles) and aligning pre-synaptic active zones (magenta, Bassoon labeling) and post-synaptic densities (yellow, Homer 1) are evident. A movie through the *z*-stack from which this maximum intensity projection was created is available (Movie EV3). Expansion factor: 9.6×. Scale bar: 1 μm.

© 2018 The Authors
