# Supplementary material for: X10 expansion microscopy enables 25‐nm resolution on conventional microscopes
Source: EMBO Rep. 2018 Jul 10;19(9):e45836. doi: 10.15252/embr.201845836 (PMC6123658; doi:10.15252/embr.201845836)
Supplement: Supplementary file 3 — Movie EV1 [file EMBR-19-e45836-s003.zip › Movie_EV1.docx]

**Movie EV1:** **3D imaging of Tubulin in COS7 cells with X10.**

The movie shows a z-scan through the Tubulin network of a COS7 cell and a rocking 3D rendering for ease of visualization. The movie is of the same frame shown in Figure 2A (scale bar: 1 µm). The movie shows the raw data images, followed by a deconvolved version.
