# Supplementary material for: X10 expansion microscopy enables 25‐nm resolution on conventional microscopes
Source: EMBO Rep. 2018 Jul 10;19(9):e45836. doi: 10.15252/embr.201845836 (PMC6123658; doi:10.15252/embr.201845836)
Supplement: Supplementary file 4 — Movie EV2 [file EMBR-19-e45836-s004.zip › Movie_EV2.docx]

**Movie EV2:** **3D imaging of peroxisomes in neurons with X10.**

The movie shows a z-scan through a group of peroxisomes (labelled for Pmp70) in primary neuronal cultures (scale bar: 1 µm). The movie shows the raw data images, followed by a deconvolved version.
