# Supplementary material for: X10 expansion microscopy enables 25‐nm resolution on conventional microscopes
Source: EMBO Rep. 2018 Jul 10;19(9):e45836. doi: 10.15252/embr.201845836 (PMC6123658; doi:10.15252/embr.201845836)
Supplement: Supplementary file 6 — Movie EV4 [file EMBR-19-e45836-s006.zip › MovieEV4/Movie_EV4_legend.docx]

**Movie EV4: 3D imaging of synapses X10.**

The movie shows a z-scan through two synapses immunostained for the synaptic vesicle clusters (identified by Synaptophysin, green), for presynaptic active zones (identified by Bassoon, magenta) and for postsynaptic densities (identified by Homer 1, yellow). The movie is of the same frame shown in Figure 4A. Scale bar: 500 nm. Movie S3 shows the raw data images, while Movie S4 shows a deconvolved version.
