# Supplementary material for: X10 expansion microscopy enables 25‐nm resolution on conventional microscopes
Source: EMBO Rep. 2018 Jul 10;19(9):e45836. doi: 10.15252/embr.201845836 (PMC6123658; doi:10.15252/embr.201845836)
Supplement: Supplementary file 8 — Movie EV6 [file EMBR-19-e45836-s008.zip › MovieEV6/MovieEV6_legend.docx]

**Movie EV6: 3D imaging of synapses in brain slices with X10.**

The movie shows a section of a brain slice stained for synaptic vesicles (green, Synaptophysin labelling), pre-synaptic active zones (magenta, Bassoon labelling), and post-synaptic densities (yellow, Homer 1 labelling). The movie is of the same frame shown in Figure EV5C. Scale bar: 830 nm. Movie S5 shows the raw data images, while Movie S6 shows a deconvolved version.
